# Acute Subdural Hematoma Without Subarachnoid Hemorrhage Due to Traumatic Aneurysm of the Posterior Cerebral Artery

**DOI:** 10.7759/cureus.96022

**Published:** 2025-11-03

**Authors:** Marika Kuboyama, Toshikazu Kimura, Airi Kugisaki, Shohei Nambu, Shunsuke Ichi

**Affiliations:** 1 Neurosurgery, Japanese Red Cross Medical Center, Tokyo, JPN

**Keywords:** acute subdural hematoma, meningeal branch, minor head trauma, posterior cerebral artery, pseudo-aneurysm

## Abstract

Acute subdural hematoma (ASDH) typically results from high-energy head trauma, such as motor vehicle accidents or severe falls. It can also be caused by minor head injuries in elderly individuals due to damage to bridging veins or small arteries, but it is considerably rarer in younger patients. A previously healthy 39-year-old man presented with progressive headaches and consciousness disturbance. The emergent computed tomography (CT) revealed ASDH covering most of the left cerebral hemisphere without subarachnoid hemorrhage (SAH). CT angiography (CTA) showed an irregularly shaped aneurysm at a distal branch of the left posterior cerebral artery (PCA) (quadrigeminal segment (P3 segment)/cortical segment (P4 segment)), suggesting that ASDH was caused by aneurysmal bleeding of the PCA. Digital subtraction angiography performed after a left decompressive craniectomy showed the aneurysm observed on CTA was suspected to be a pseudo-aneurysm, and parent artery occlusion was performed. This report presents a rare case of ASDH in a young individual without a history of head trauma. Our case highlights the importance of considering aneurysm rupture as a potential etiology in patients with ASDH without SAH, particularly in the absence of a history of head trauma.

## Introduction

Acute subdural hematoma (ASDH) typically results from high-energy head trauma, such as motor vehicle accidents or severe falls. Approximately two-thirds of ASDH cases result from large contusions bleeding into the subdural space, while the remainder are caused by disruption of superficial bridging veins connecting the cortex to the venous sinuses [1]. ASDH induced by bridging veins tends to be found in elderly patients with a much higher incidence rate compared to younger patients [1]. Besides ASDH resulting from severe head injuries, ASDH can occur in elderly individuals due to low-impact injuries. In elderly individuals, brain atrophy [2] and decreased elasticity of the cerebral vasculature make them more susceptible to the shearing forces that lead to ASDH formation even with seemingly minor trauma. In younger individuals, ASDH from minor trauma is considerably rare, and cases associated with pseudo-aneurysms have rarely been reported [3].

Though ASDH is usually caused secondary to head trauma, it also results from intracerebral hemorrhages due to various causes, including ruptured intracranial aneurysms, arteriovenous malformations, and coagulopathy [4]. Convexity meningiomas and other superficial neoplasms have also been described as rare causes of subdural hematomas (SDHs) [5]. ASDH caused by ruptured aneurysms without subarachnoid hemorrhage (SAH) is particularly uncommon and can pose a diagnostic challenge, especially when the aneurysm is distant from the hematoma [6,7].

This report presents a rare case of ASDH in a young patient without a history of head trauma, initially mimicking a non-traumatic cause, ultimately due to aneurysmal bleeding later identified as a pseudo-aneurysm.

## Case presentation

A 39-year-old man with no significant medical history presented with progressive headaches and subsequent consciousness disturbance. Initially, he experienced headaches and fever, and tested positive for severe acute respiratory syndrome coronavirus 2 (SARS-CoV-2). However, his headache worsened the following day, and he was transported to our hospital after rapidly deteriorating to a coma upon contact with the ambulance.

**Figure 1:**
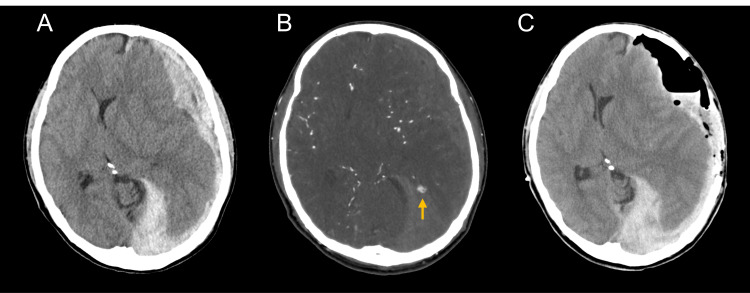
An acute subdural hematoma and an aneurysm at the quadrigeminal segment (P3 segment)/cortical segment (P4 segment) of the left posterior cerebral artery Initial emergent computed tomography (CT) scan showed an acute subdural hematoma covering a large area of the left cerebral hemisphere and uncal herniation without subarachnoid hemorrhage (A). CT angiography revealed an irregularly shaped aneurysm at the quadrigeminal segment (P3 segment)/cortical segment (P4 segment) of the left posterior cerebral artery (arrow (B)). The CT scan performed immediately after the decompressive craniectomy showed that most of the frontotemporal hematoma was removed (C).

Upon arrival, his Glasgow Coma Scale (GCS) was E1V1M2 with right-sided hemiparesis, and his pupils were anisocoric with no light reflex. Emergent computed tomography (CT) of his head revealed an ASDH covering most of the left cerebral hemisphere, resulting in uncal herniation (Figure 1A). No SAH was detected; however, due to the time course of the headache and absence of any history of severe head injury, CT angiography (CTA) was performed to identify the source of the bleeding. It revealed an irregularly shaped aneurysm at a distal branch of the left posterior cerebral artery (PCA) (quadrigeminal segment (P3 segment)/cortical segment (P4 segment)) (Figure 1B). Thus, ASDH was supposed to be caused by a ruptured aneurysm of the PCA. At first, a left decompressive craniectomy and hematoma removal were urgently performed to relieve the uncal herniation (Figure 1C). Subsequently, digital subtraction angiography was performed for coil embolization of the aneurysm.

**Figure 2:**
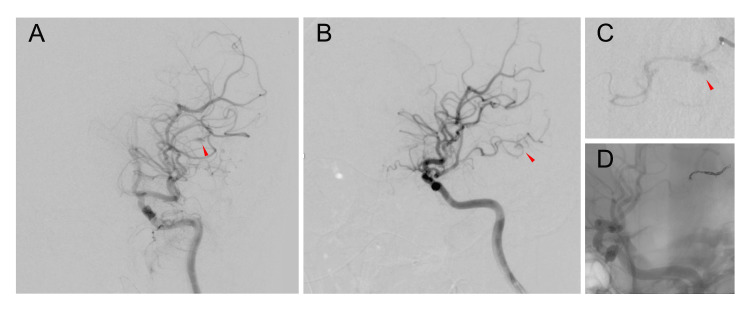
Pseudo-aneurysm detected in the digital subtraction angiography The anteroposterior (A) and lateral (B) views of the digital subtraction angiography of the left internal carotid artery revealed the aneurysm (arrowheads). The irregularly shaped aneurysm observed on computed tomography angiography was a pseudo-aneurysm (C, arrowhead), and the parent artery occlusion was performed (D).

The irregularly shaped aneurysm observed on CTA was suspected to be a pseudo-aneurysm based on the results of cerebral angiography (Figure 2A-2C), and parent artery occlusion was performed (Figure 2D). A subcutaneous hematoma suggestive of a minor head injury was also observed on the patient’s vertex during the craniectomy. The transthoracic echocardiography identified no cardiac vegetation, and the results of the three sets of blood culture were negative. His initial laboratory tests showed a marked decrease in platelets and elevated serum levels of hepatic enzymes and gamma-glutamyl transferase (GGT) (Table 1).

**Table 1:** The initial laboratory results after the arrival at our hospital

Parameters	Results	Reference Range
Platelet count	5.8×10^4^ /μL	13.0×10^4^-40.0×10^4^ /μL
Prothrombin time-international normalized ratio (PT-INR)	1.02	0.90-1.10
Aspartate aminotransferase (AST)	129 U/L	13-20 U/L
Alanine aminotransferase (ALT)	54 U/L	10-42 U/L
Gamma-glutamyl transferase (GGT)	324 U/L	13-64 U/L
Total bilirubin	1.2 mg/dL	0.4-1.5 mg/dL

It was suspected that he had alcoholic liver disease, which caused the decrease in platelets. After receiving medical treatment for the acute phase and undergoing cranioplasty and rehabilitation at our hospital, his consciousness was GCS E4VTM4 with the Glasgow Outcome Scale of 3 (Severe Disability). He remained dependent on others for some activities of daily living and was subsequently transferred to a neurorehabilitation hospital.

## Discussion

A case of ASDH without SAH resulting from a pseudo-aneurysm of the distal PCA was presented. Traumatic intracranial aneurysms in the PCA are rarely reported. Komiyama et al. reviewed 171 cases of traumatic aneurysms reported since 1960 [8]. Traumatic intracranial aneurysms in the posterior circulation were detected in only 8% of the cases, with only two cases involving the PCA [8]. In another literature review of the 11 cases involving traumatic PCA aneurysms, more than half of the aneurysms were found to be located in the precommunicating segment (P1 segment) and postcommunicating segment (P2 segment) of the PCA [9]. In this report, the injuries were primarily attributed to high-energy incidents such as car accidents or nasty falls, with only one case involving a traumatic aneurysm resulting from a bicycle accident [9].

Among cases of ASDH resulting from bleeding due to traumatic aneurysms, only a few cases are attributed to minor head traumas. One such case involved a minor bicycle crash [10]. The pseudo-aneurysm was identified in the P3/4 segment of the PCA, similar to our case [10]. Another instance occurred when a patient struck their occipital region against a mat at home, leading to a pseudo-aneurysm in the left posterior temporal artery [11]. In the current case, minor head trauma was suspected because the irregularly shaped aneurysm observed on CTA was identified as a pseudo-aneurysm on cerebral angiography, and a subcutaneous hematoma was found during the craniectomy.

In terms of the mechanism of the formation of the pseudo-aneurysm in our case, we hypothesize that it may have occurred due to damage to a branch of the PCA extending to the tentorium cerebelli, similar to the artery of Davidoff and Schechter (ADS), which is a meningeal branch of the P1 or P2 segment of the PCA [12]. In such a scenario, the presence of the pseudo-aneurysm in the P3/4 segment of the PCA in our case would be plausible. ADS supplies the dura of the inferomedial part of the tentorium cerebelli and is associated in hemodynamic balance with the artery of Bernasconi and Cassinari [12], which is the tentorial artery originating from the internal carotid artery [13]. ADS was initially described by Wollschlaeger and Wollschlaeger in 1965, who identified it in 90% of cadaveric dissections through postmortem angiography, with subsequent in vivo discovery by Weinstein et al [14]. A tentorial branch, originating from the rostral trunk of the superior cerebellar artery (SCA) to the inferior aspect of the tentorium in its free edge, has also been documented [15]. In a study involving patients who underwent microvascular decompression for trigeminal neuralgia, the meningeal branch of the SCA was observed in 15 out of 58 patients (25.9%) [16]. We postulate that minor head trauma caused shear stress on a meningeal branch from the distal PCA, leading to the formation of the pseudo-aneurysm. This explanation is supported by the absence of cardiac vegetations on transthoracic echocardiography and negative blood cultures, making an infectious or pre-existing aneurysm unlikely.

Natural hemostasis may have been impaired due to the patient's alcoholic liver disease. Liver dysfunction can cause thrombocytopenia, platelet dysfunction, and reduced synthesis of clotting factors, increasing bleeding risk [17] and the risk of hematoma enlargement [18]. Both alcoholic liver cirrhosis and non-cirrhotic alcoholic liver disease have been reported as risk factors for intracerebral hemorrhage [19]. In this case, we propose that the hematoma resulting from bleeding of the pseudo-aneurysm after the minor trauma was exacerbated by the patient's liver dysfunction.

## Conclusions

In our case, ASDH without SAH was supposed to be due to rupture of the pseudo-aneurysm of a distal branch of the PCA caused by the minor head injury, though there wasn’t an apparent history of head trauma. The meningeal branch of the PCA supplying the tentorium cerebelli was supposed to be damaged and torn by a slight impact to the head. This rare but critical clinical scenario in our case underscores the importance of considering pseudo-aneurysm as a potential etiology in patients presenting with pure SDH even without SAH, in the absence of an obvious history of head trauma. This case also highlighted the importance of performing cerebrovascular assessments when a hematoma was detected with atypical clinical courses or images.
